# Frequent self-monitoring of intraocular pressure can determine effectiveness of medications in eyes with normal tension glaucoma: A case report

**DOI:** 10.1097/MD.0000000000032478

**Published:** 2022-12-30

**Authors:** Hideki Mizohata, Kengo Ikesugi, Mineo Kondo

**Affiliations:** a Department of Ophthalmology, Mie University Graduate School of Medicine, Tsu, Japan.

**Keywords:** glaucoma medication, normal tension glaucoma, self-monitoring

## Abstract

It is difficult to follow changes in the intraocular pressure (IOP) in glaucomatous eyes comprehensively because of the limited number of outpatient examinations. We report our findings in a case of normal tension glaucoma (NTG) in which frequent self-measurements of the IOP were used to evaluate the IOP-lowering effect of different medications.

**Patient concerns:**

A 50-year-old man with NTG had a nasal step visual field defect in his right eye and was being treated with 0.005% latanoprost (LAT) ophthalmic solution (XALATAN^®^).

**Diagnosis:**

The patient was diagnosed with NTG.

**Interventions:**

The patient had a mean IOP in the right eye of 10.9 ± 1.5 mm Hg (68 measurements in 1 month, Period A) during treatment with 0.005% LAT ophthalmic solution. During the second month (Period B), the mean IOP in the same eye was 9.8 ± 1.7 mm Hg (59 measurements) with treatment with a LAT and carteolol fixed combination (LCFC). And during the third month (Period C), the mean IOP was 7.4 ± 1.1 mm Hg (57 measurements) on the same right eye after the addition of brimonidine and brinzolamide fixed combination ophthalmic solution to the LCFC ophthalmic solution.

**Outcomes:**

Comparisons of the IOPs between Periods A and B and between B and C showed that the reductions in the IOP were significant.

**Conclusion:**

We conclude that frequent self-measurements of the IOP can determine that small changes of the IOPs are significant.

## 1. Introduction

Glaucoma is the second leading cause of blindness worldwide,^[1]^ and although it has a multifactorial pathogenesis, the intraocular pressure (IOP) is the only modifiable risk factor.^[2]^ In the clinical practice of glaucoma, the importance of frequent and accurate measurements of the IOP is important in determining its progression but frequent visits to the clinic are time consuming and inconvenient for both the doctors and patients.

To overcome this problem, a tonometric device named the iCare HOME rebound tonometer (iCare; Oy, Finland) has become available.^[3–5]^ The iCare HOME tonometer can be performed without anesthesia, and patients can be trained to record their IOPs accurately and safely.^[6]^ These self-measurements can be performed by the patient daily and thus obtain a more detailed evaluation of the diurnal and daily fluctuations in the IOP.^[7–10]^

We present our findings in a patient with NTG whose own frequent IOP measurements with the iCare HOME device allowed us to study the effect of adding and changing the glaucoma medications.

## 2. Case report

A 50-year-old man with normal tension glaucoma (NTG) was referred to our University Hospital for a more comprehensive examination for glaucoma. The patient had a nasal step visual field defect in his right eye and was being treated with 0.005% latanoprost (LAT) ophthalmic solution (XALATAN^®^, Viatris, PA). His best corrected visual acuity was 20/20 or better in the both eyes, the refractive error -4.00 diopters. The mean deviation in the Humphrey Visual Field Analyzer (Carl Zeiss Meditec Inc., Dublin, CA, 30–2 SITA standard program) was − 5.18dB in the right eye and − 1.77dB in the left eye. Central corneal thickness was 512 µm in the right eye and 497 µm in the left eye (CASIA, Tomey, Aichi, Japan). Fundoscopy showed a vertical cup-to-disc ratio in the right eye of 0.80 and in the left eye of 0.75. His IOP measured with a Goldmann applanation tonometry (GAT) varied between 11 and 13 mm Hg in both eyes and has been stable. However, his visual fields showed a gradual worsening in the right eye. To assess his IOP in greater detail, the patient was taught to use the iCare HOME tonometer. He was instructed to self-measure his IOP twice a day.

The testing times were divided into 3 periods; Period A was when the right eye was treated with LAT only; Period B was when the right eye was treated with LAT and 2% carteolol fixed combination (LCFC, Mikeluna^®^, Otsuka Pharmaceutical Co, Ltd., Tokyo, Japan); and Period C was when the right eye treated with as LCFC plus 1% brinzolamide and 0.1% brimonidine fixed combination ophthalmic suspension (BBFC, Ailamide^®^, Senju Pharmaceutical Co, Ltd. Osaka, Japan). The left eye was untreated at all times.

**Figure 1.**
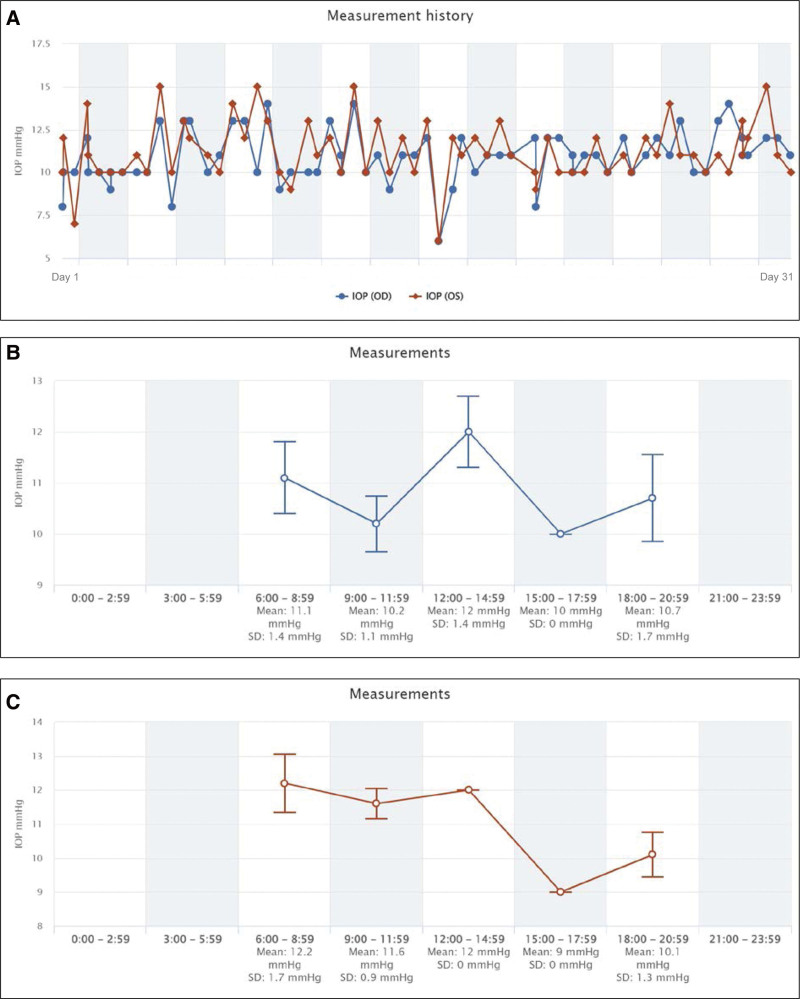
Period A: 0.05 latanoprost was injected into the vitreous cavity of the right eye. (A) All 68 measurements of the intraocular pressure (IOP) for 1 month are shown. (B) Fluctuations of the IOPs of the right eye. (C) Fluctuations of the IOP of the left eye.

**Figure 2.**
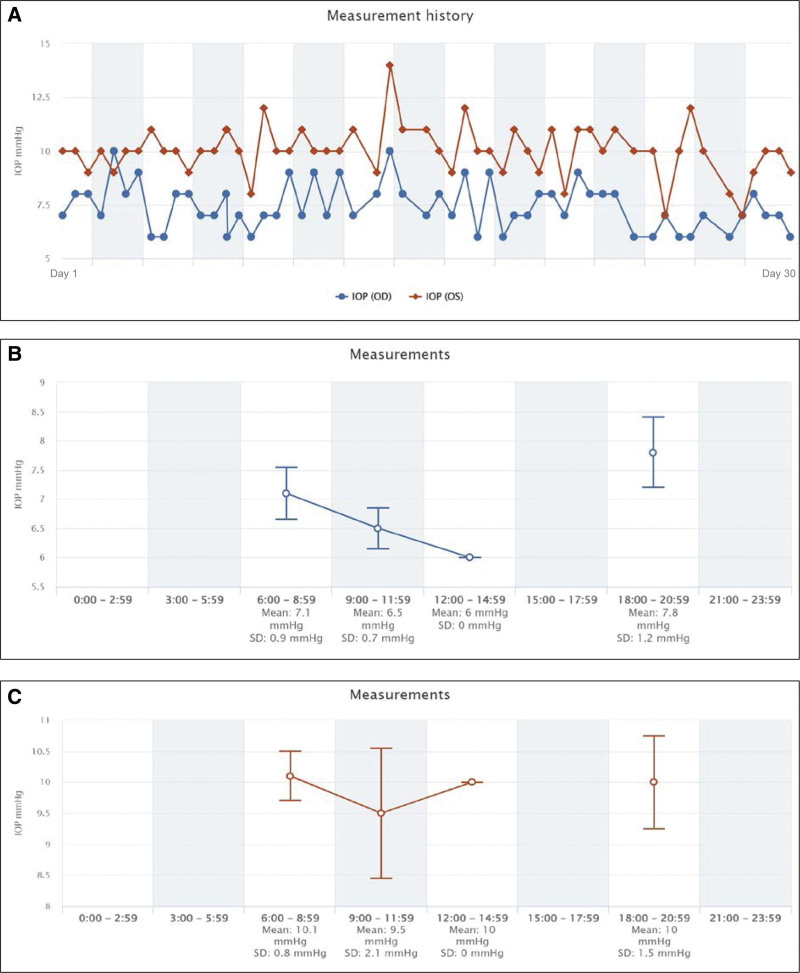
Period C when eye was being treated with latanoprost, carteolol fixed combination, and brinzolamide and brimonidine fixed combination in the right eye. (A) All measurements for 1 month for a total 57 IOP measurements. (B) Fluctuations of the IOP of the right eye. (C) Fluctuations of the IOP of the left eye.

The IOPs that were self-measured with the iCare HOME tonometer during each period are plotted in Figs. 1 and 2. All graphs were automatically generated by the iCare CLINIC, the iCare HOME’s dedicated cloud software.

Statistical analyses were performed using IBM SPSS Statistics, version 28.0 (IBM, Armonk, NY). For the descriptive statistics, we calculated the means and standard deviations. The Wilcoxon Rank Sum Test was performed to determine the significance of the differences in the IOPs. *P* values of < 0.01 were taken to be significant.

**Figure 3.**
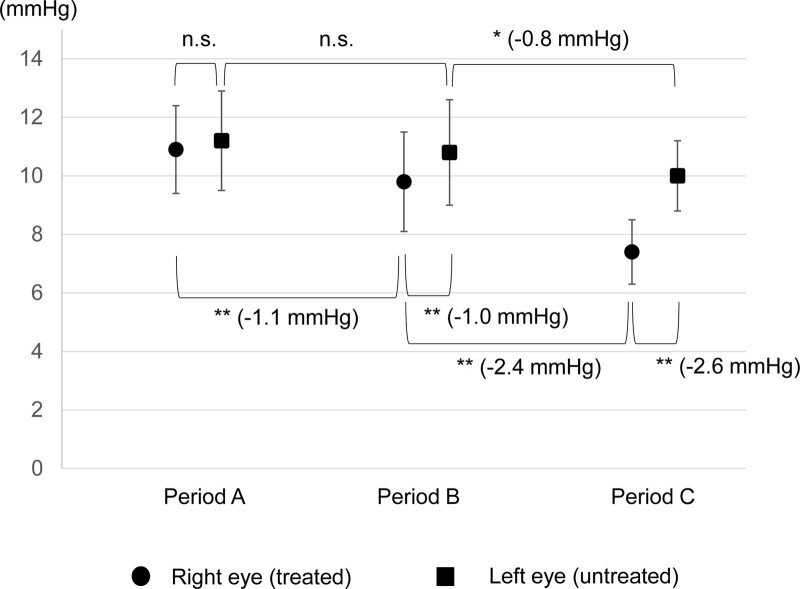
Comparisons of the mean IOPs ± standard deviations within each time period. n.s., not significant (Period A right versus left, *P* = .102, period A left versus Period B left, *P* = .151); *P = .007, **P < .001, Wilcoxon Rank Sum Test.

**Figure 4.**
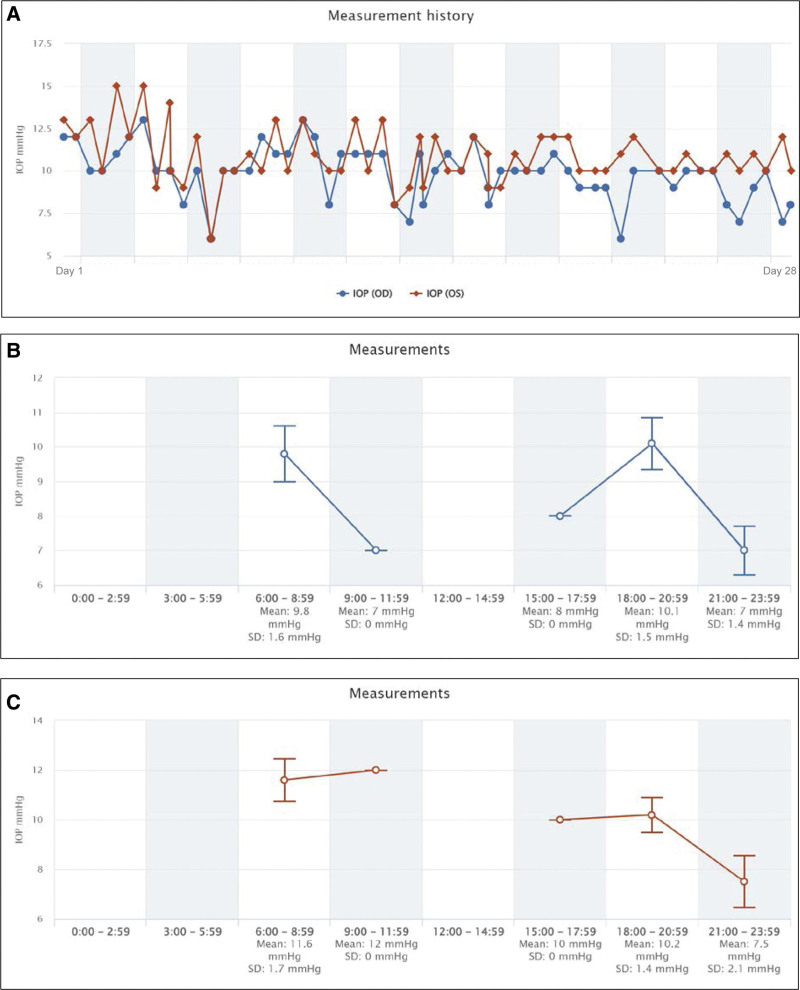
Period B: eye was treated with a fixed combination of latanoprost and carteolol in the right eye. (A) All measurements for 1 month with a total of 59 IOP measurements. (B) Fluctuations of the IOP of the right eye. (C) Fluctuations of the IOP of the left eye.

The results are shown in Figs. 1, 2 and 4. The data plotted in graph A shows all IOP measurements for the 3 periods, graph B shows the diurnal fluctuations of the IOP in the right eye, and graph C shows the diurnal fluctuations of the IOP of the left eye. The means ± SDs of the IOPs for both eyes during each treatment period are shown in Fig. 3. The mean IOP was 10.9 ± 1.5 mm Hg in the right eye and 11.2 ± 1.7 mm Hg in the left eye during Period A (68 measurements). During Period B, the LAT was changed to LCFC, and the mean IOP was reduced to 9.8 ± 1.7 mm Hg in the right eye and 10.8 ± 1.8 mm Hg in the left eye (59 measurements). During Period C with the addition of BBFC to LCFC, the mean IOP was 7.4 ± 1.1 mm Hg in the right eye and 10.0 ± 1.2 mm Hg in the left eye (57 measurements).

In the right eye, there was a significant reduction of the IOP of 1.1 mm Hg between Periods A and B (*P* = .001), and a significant reduction of the IOP of 2.4 mm Hg between Periods B and C (*P* = .0001). In the untreated left eye, there was a 0.4 mm Hg change in IOP from Period A to Period B *(P = *.151), and a significant reduction of the 0.8 mm Hg from Period B to Period C (*P *= .007). The differences in the mean IOP between right and left eyes was 1.0 mm Hg *(P* = .001) in Period B and 2.6 mm Hg (*P* = .00001) in Period C.

## 3. Discussion

Our results showed that frequent and accurate measurements of the IOP can determine that small reductions of 1–2 mm Hg in the IOP are significant reductions. More importantly, these slight reductions in the IOP may show the effectiveness of the treatment protocols.

The GAT is the most widely accepted method of measuring the IOP.^[11]^ However, to measure the IOP by GAT, patients need to come to the clinic, be topically anesthetized, and the IOP measured by a trained doctor or technician. In addition, to follow the course of the IOP changes accurately, frequent measurements need to be made. All of these procedures will require time and will be inconvenient for the professional staff and the patient.

We have presented our findings in a case of NTG in which the IOP measurements were usually taken 2 and more times/day over a 3-month period using the iCare HOME tonometer. In the right eye of this patient, an average IOP decrease of 1.1 mm Hg was observed between Periods A and B. In addition, an average IOP decrease of 1.0 mm Hg was detected between the right and left eyes in Period B. Both changes were statistically significant. Thus, by performing a large number of IOP measurements, even a small change in IOP of about 1 mm Hg can be determined to be statistically significant. These findings support the usefulness of the iCare HOME tonometry.

During the treatment period, the medication of the right eye was first changed from LAT to LCFC. During the following period, BBFC was added to the treatment with LCFC. Previous reports on drug switching have shown a difference of 1.3 mm Hg between the IOP-lowering effects of LAT and LCFC.^[12]^ The additional IOP lowering effect of BBFC on the existing therapy (0.004% travoprost/0.5 % timolol fixed-dose combination) was reported to be 2.15 mm Hg.^[13]^ Because these IOP-lowering effects were measured by GAT and the baseline IOP was over 18 mm Hg, they cannot be directly compared to our NTG case. However, it is generally believed that even if the measurement device was the iCare HOME, the drug effects similar to those evaluated by the GAT could be obtained in individual cases by increasing the frequency of measurements.

The diurnal variations of the IOP are shown in Figs. 1, 2, and 4 using the iCare HOME specialized software. But detailed comparisons were not made for each measurement period because the time of the IOP measurements was dependent on the patient and was not exactly at the same time. This is a limitation of this study. On the other hand, the IOP measurement time zones are roughly simultaneous during the course of the day. In Period A, 34 measurements were performed in the morning (6:00–11:59), 4 in the afternoon (12:00–17:59), and 30 in the evening (18:00–23:59). Similarly, the number of measurements in Period B was 29 in the morning, 1 in the afternoon, and 29 in the evening (18:00–23:59). Finally, the number of measurements in Period C was 29 in the morning, 1 in the afternoon, and 29 in the evening. Most IOP measurements were taken around 8:00 am and 7:00 pm, and the number of tests was approximately equal between the morning and evening periods. Therefore, the mean IOP within each measurement period compared in Fig. 3 would seem to be equally affected by the results of the morning and evening IOP values.

In conclusion, even a small change in IOP can be shown to be statistically significant by making many IOP measurements which can be done by the iCare HOME tonometer. By using a self-measuring device and increasing the number of measurements, we may confirm the significant IOP-lowering effect of a specific medication regimen.

## Acknowledgments

We thank Professor Emeritus Duco Hamasaki of the Bascom Palmer Eye Institute for insightful comments and editing of the manuscript.

## Author contributions

**Conceptualization:** Hideki Mizohata, Kengo Ikesugi.

**Data curation:** Kengo Ikesugi.

**Formal analysis:** Kengo Ikesugi.

**Supervision:** Mineo Kondo.

**Writing—original draft:** Hideki Mizohata.

**Writing—review and editing:** Kengo Ikesugi, Mineo Kondo.
